# Staged resection in the management of HIV-related anogenital giant condyloma acuminatum. A case report

**DOI:** 10.1016/j.amsu.2019.10.024

**Published:** 2019-11-01

**Authors:** Guo Hou Loo, Li Yi Lim, Zulkifli Md Zainuddin, Xeng Inn Fam

**Affiliations:** Department of Surgery, Universiti Kebangsaan Malaysia Medical Centre, Jalan Yaacob Latiff, Bandar Tun Razak, 56000, Kuala Lumpur, Malaysia

**Keywords:** Bushke-lowenstein tumour, Human immunodeficiency virus, Human papilloma virus, Anogenital warts, Defunctioning colostomy in genital warts

## Abstract

**Introduction:**

Giant condyloma acuminata (GCA), also known as Bushke-Lowenstein tumour, is a rare disease which affects 0.1% of the population. Although histopathologically benign, it tends to be locally destructive. The common sites of involvement include the penis and the anorectum. Due to the rarity of the disease, there is a lack of controlled studies on the optimal management of this entity. Thus, we report a case of anogenital GCA in a 40-year-old HIV-positive man.

**Case presentation:**

A 40-year-old man presented with progressive anogenital warts associated with foul-smelling discharge and fever. He has been diagnosed with HIV and was on HAART on presentation. A warty excrescence had infiltrated the entire external genitalia, gluteals and sacral region. Serial excision was performed along with a defunctioning colostomy. The patient recovered well, and the final histopathological showed features of GCA.

**Discussion:**

With HIV, the HPV infection goes unchecked may develop into GCA. Malignant transformation to squamous cell carcinoma may occur in more than half of the cases. A complex interaction between HIV and HPV may lead to a higher risk of recurrence even after resection. The diagnosis is usually clinical. Imaging modalities may be used in identifying the extent and depth of invasion.

**Conclusion:**

The optimal management of anogenital giant condyloma acuminata remains to be determined. Staged surgical excision should be conducted to achieve an optimum outcome. Radical reconstructive surgery should be reserved for patients with recurrence, malignant transformation or sphincter involvement.

## Highlights

• Giant condyloma acuminata (GCA) is a rare disease which is locally destructive in nature.
• Patients usually delay treatment until the growth restricts mobility or when it becomes infected.
• With HIV co-infection, the HPV infection goes unchecked may develop into GCA.
• Malignant transformation to squamous cell carcinoma may occur in more than half of the cases.
• Staged surgical excision with R0 resection should be conducted to achieve an optimum outcome.

## 1 Introduction

Giant condyloma acuminata (GCA), also known as Bushke-Lowenstein tumour, is a rare disease which affects 0.1% of the population [1]. It is considered as an intermediate step between the condyloma acuminata and squamous cell carcinoma and may represent a variant of verrucous carcinoma [1,2]. It is commonly associated with human papillomavirus type 6 and type 11 infection. Although histopathologically benign, it tends to become very large, often exceeding 10cm in greatest diameter, and locally destructive [3].

The most common sites of involvement include the penis and the anorectum; however, it may also involve the groin, scrotum and the pubis [3]. It tends to grow both exophytically and endophytically, and the exophytic outgrowths have been described as “cauliflower-like”, with a propensity for inter-papillary fistulation [3]. Malignant transformation may occur in up to 56% of cases, and there is a high risk of recurrence after surgical resection [4]. It is three times more common in men, and usually affects men below the age of 50years [3,5].

Immunosuppression, tobacco smoking, and early age of sexual intercourse seem to confer an increased risk of developing GCA [6]. Due to the rarity of the disease, there is a lack of controlled studies on optimal management. With that in mind, we report a case of GCA in a 40-year-old HIV-positive man who presents with anogenital GCA. He underwent serial debridement, and during the last follow-up, he remains well, with no signs of disease progression. This case has been reported in line with the SCARE criteria [7].

## 2 Case presentation

A 40-year-old man presented to us with progressive anogenital warts associated with foul-smelling discharge and fever. It was initially small in size and confined to the penile foreskin; however, over the past three years, it grew considerably. The sheer size of the anogenital warts made it impossible for him to ambulate or maintain perineal hygiene. He had been diagnosed with HIV for the past three years and was on HAART on presentation. His CD4^+^ count was 159 cells/uL and HIV RNA was 38 copies/mL upon presentation.

**Fig. 1:**
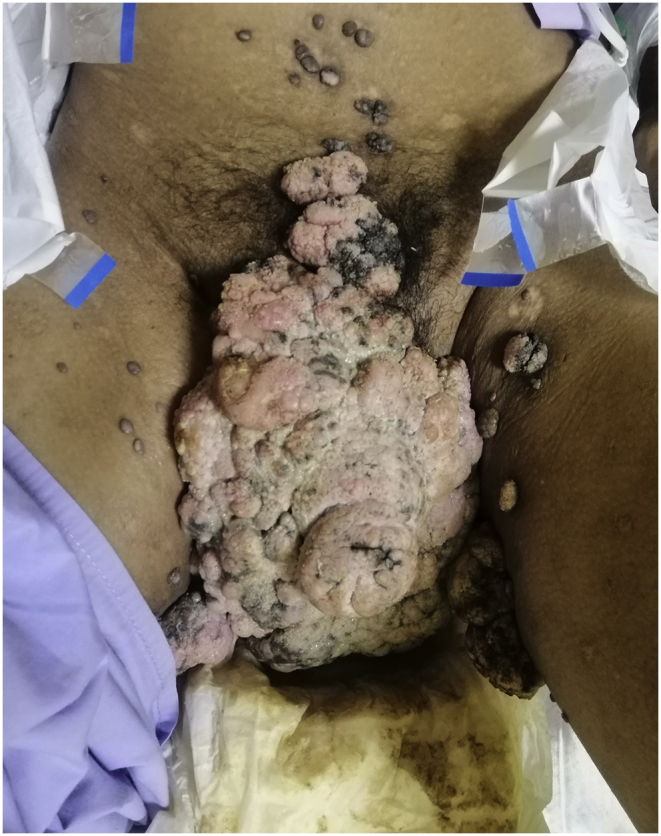
Anterior view of the groin upon presentation. Warty excrescence is seen covering the entire external genitalia.

**Fig. 2:**
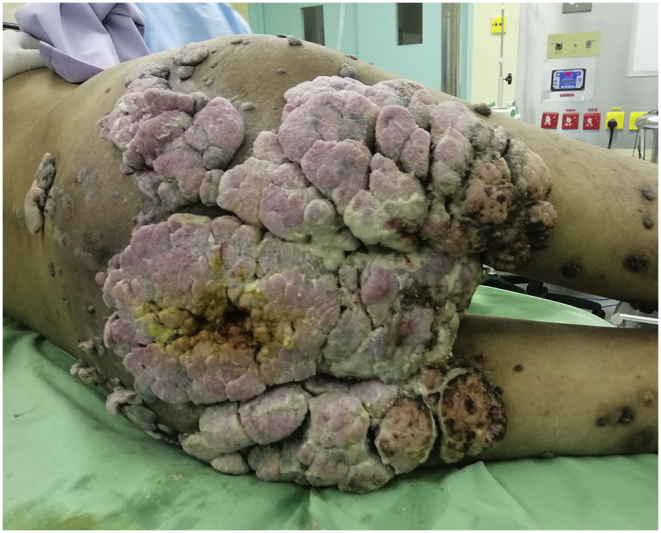
Posterior view of the gluteal and sacral region (patient on left lateral position). GCA can be seen invading his entire gluteal and sacral region, with faecal soiling adjacent to the anal opening.

On examination, he was febrile with a strong malodorous stench emitted from his anogenital warts. The exophytic warty excrescence had infiltrated the entire external genitalia making his penis, scrotum and external urethral meatus indiscernible (Fig. 1). Posteriorly, warts invaded his entire gluteal and sacral region, and a vague anal opening could be perceived based on faecal soiling over that region (Fig. 2). He denied any lower urinary symptoms but admitted for faecal incontinence. He was started on broad-spectrum intravenous antibiotics, and he was brought to operating theatre for excision of GCA. Serial excision of the GCA was performed as each time, we encountered torrential bleeding from the warts (estimated blood loss was 1.5 L–2.5 L), and we had to stop the surgery. A transverse defunctioning colostomy was performed as well to prevent faecal wound contamination. Daily dressings were performed for the wounds to allow secondary healing.

**Fig. 3:**
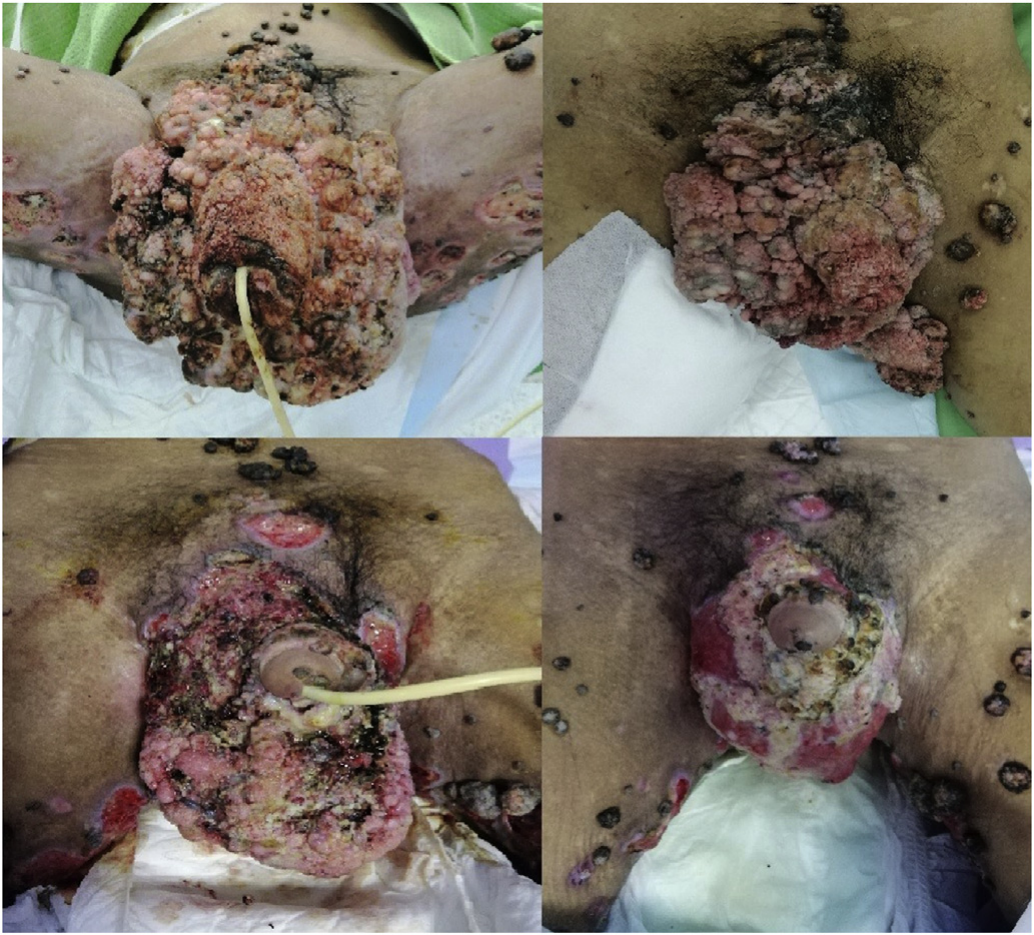
Serial images of anterior view of the groin region after serial excision.

**Fig. 4:**
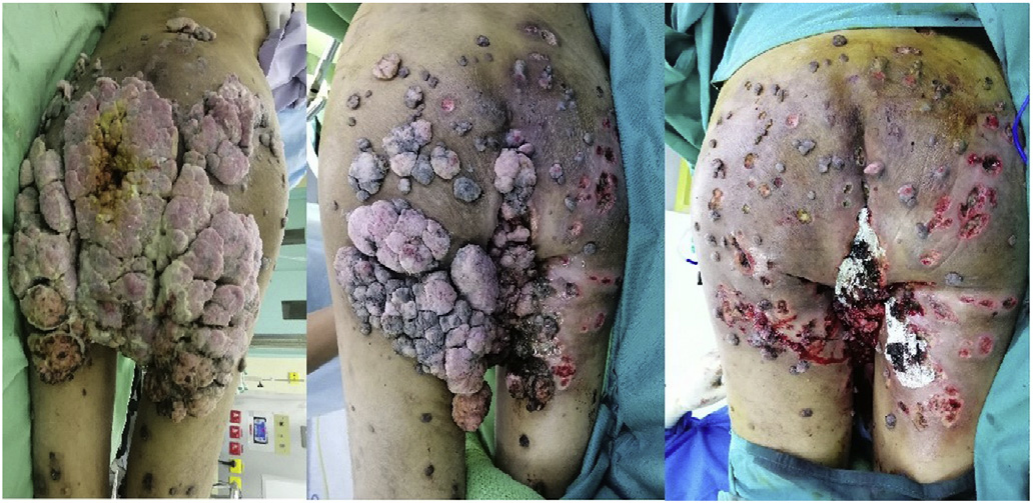
Serial images of the gluteal and sacral region after serial excision.

We were able to reduce the size of the GCA considerably, and only a small area of warts was left behind over his scrotal skin (Fig. 3, Fig. 4). After a multidisciplinary meeting, we decided to reserve reconstructive surgery for recurrence, and further excision was not performed. The patient recovered well with oral nutritional support, dressings and physiotherapy, and he was able to ambulate upon discharge. The histopathology of the specimen showed features suggestive of GCA with extensive koilocytes and was negative for p16 immunostaining.

At the last evaluation, he was well and able to maintain his perineal hygiene. The GCA did not progress significantly, and there were no signs of infection. He was comfortable with his present state and did not wish for further surgical intervention.

## 3 Discussion

Condyloma acuminata (genital warts) is a benign papillary epithelial lesion, caused by the Human Papilloma Virus infection [8]. It is the most common sexually transmitted disease among young adults, and common affected sites include the external genitalia, anus and surrounding areas [9]. Giant condyloma acuminata (GCA), also known as Bushke-Lowenstein tumour, is a rare disease which affects 0.1% of the population [1]. Immunosuppression, tobacco smoking, and early age of sexual intercourse seem to confer an increased risk of developing GCA [6]. It is three times more common in men, and usually affects men below the age of 50years [3,5].

Under specific situation such as HIV infection, diabetes, malignancy, post-transplantation and pregnancy, the HPV 6 and 11 infection goes unchecked and develops into GCA [10]. Malignant transformation to squamous cell carcinoma may occur in more than half of the cases, and it is more commonly seen in HIV-infected patients [4]. HIV enhances the HPV transcription and upregulates HPV E7, which in turn leads to an increased HPV DNA in infected tissues [11]. Local immune control by Langerhans cells, macrophages and CD4 cells are affected due to a reduction in their quantities by HPV [11]. This complex interaction between HIV and HPV may lead to a higher risk of recurrence even after surgical resection [10].

The most common presenting complaint is a slow-growing, fungating, “cauliflower-like” mass over the external genitalia and perianal region [4]. This is commonly associated with pelvic pain, secondary infections with purulent discharge and bleeding [8]. Less common associated symptoms include faecal incontinence, weight loss, difficulty in ambulation, anaemia with fatigue, perianal itch, haemorrhoids, dysuria, and abdominal distension [4].

The diagnosis is usually clinical. Polymerase chain reaction for HPV DNA has been used to confirm the type of HPV infection [12]. The role of imaging modalities such as the MRI or CT is to assist in identifying the extent and depth of invasion. It may also serve to delineate any involvement of vital structures, such as the anal sphincter, especially in perianal GCA [1]. This is important when considering the feasibility and extent of surgical resection [1]. In our case, we did not perform any preoperative imaging as clinically we did not suspect pelvic invasion or sphincter involvement.

The differential diagnosis for GCA is verrucous carcinoma, with some authors advocating both entities are part of a continuous spectrum [5]. However, GCA always harbours HPV while anogenital verrucous carcinoma does not [3]. Although on clinical examination both exhibit similar characteristics, the warty excrescence of GCA appears more delicate and have a softer texture when compared to verrucous carcinoma [3]. On histopathological examination, a key feature distinguishing GCA from verrucous carcinoma is the presence of koilocytes [3]. It is important to note that both GCA and verrucous carcinoma exhibit negative or focal immunostaining with p16 [3].

Various modalities of treatment exist, with surgical resection being the most commonly employed, especially during the first presentation [13]. Wide local excision seems to be the treatment of choice during the early phase of the disease, with reconstructive and more radical surgeries reserved for recurrent cases [14]. The aim of surgery should be complete surgical removal of GCA, with optimal preservation of normal tissue function [15]. A loop colostomy may be performed to prevent wound contamination [15,16]. When there is extensive involvement of various structures such as in our case, we preferred a staged surgical resection. This approach will reduce the requirement of blood products, shorten the operative time, and allow a controlled dissection of the tumour. Abdominoperineal resection is recommended in cases of GCA with pelvic involvement [15]. In patients with recurrent, inoperable disease or those patients not fit for surgery, chemotherapy and radiotherapy may have a role [1,16]. Despite various modes to treatment, the overall recurrence rate is reported to be 67%, with the overall mortality rate being 21% [4].

## 4 Conclusion

The optimal management of anogenital giant condyloma acuminata remains to be determined. Staged surgical excision should be conducted to achieve an optimum outcome. Radical reconstructive surgery should be reserved for patients with recurrence, malignant transformation or sphincter involvement.

## Ethical approval

Ethical approval has been exempted by our institution's ethics committee (The National University of Malaysia's Ethics Committee) as this publication is a case report, provided that patients/patient's next-of-kin have given their informed written consent for the publication of this case report.

## Sources of funding

No source of funding.

## Author contribution

Study concepts: Loo Guo Hou.

Study design: Data acquisition: Loo Guo Hou.

Quality control of data and algorithms: Loo Guo Hou.

Data analysis and interpretation: Loo Guo Hou.

Statistical analysis: Not applicable.

Manuscript preparation: Loo Guo Hou.

Manuscript editing: Loo Guo Hou, Fam Xeng Inn, Zulkifli Md. Zainuddin.

Manuscript review: Fam Xeng Inn, Zulkifli Md. Zainuddin.

## Trial registry number

Not applicable.

## Guarantor

Loo Guo Hou, Fam Xeng Inn.

## Consent

Written informed consent was obtained from patient and patient's next of kin for publication of this case report and accompanying images. A copy of the written consents is available for review by the Editor-in-Chief of this journal on request.

## Provenance and peer review

Not commissioned, editor reviewed.

## Declaration of competing interest

No conflict of interests.
